# Incidence of Open Gingival Embrasure During Orthodontic Treatment and Associated Factors: A Systematic Review

**DOI:** 10.1155/ijod/1731448

**Published:** 2026-08-04

**Authors:** Charles Angioni, Marwa Fendi, Zeyad Hassan, Agnès Kamoun, Marjolaine Gosset, Adrian Brun

**Affiliations:** ^1^ Division of Dentistry, Faculty of Health, Paris Cité University, Paris, France, ciup.fr; ^2^ Oral Medicine Department, Henri Mondor Hospital, APHP, Créteil, France, aphp.fr; ^3^ Oral Medicine Department, Bretonneau Hospital, APHP, Paris, France, aphp.fr; ^4^ Oral Medicine Department, Rothschild Hospital, APHP, Paris, France, aphp.fr; ^5^ Inserm, Oral Health, UMR-S 1333, Paris Cité University and Sorbonne Paris Nord, Paris, France, inserm.fr

**Keywords:** associated factor, incidence, interdental papilla, orthodontics

## Abstract

**Aim:**

The aim of the present systematic review is to assess the incidence of open gingival embrasure (OGE) and associated factors during orthodontic treatment in individuals in permanent and healthy dentition.

**Material and Methods:**

This systematic review followed Preferred Reporting Items for Systematic Reviews and Meta‐Analyses (PRISMA) guidelines. Electronic databases (Medline, Cochrane, Embase, Scopus) were searched through July 2025 to answer the following PEO question: ’In individuals in permanent and healthy dentition, what is the incidence of interdental OGE during orthodontic treatment and what are the associated factors?’ (Prospero: CRD420251084329). The eligibility criteria included clinical studies assessing the incidence of OGE during orthodontic treatment in patients in permanent dentition without periodontitis. A qualitative synthesis was undertaken by systematically summarising the characteristics of the studies, as well as the demographic, periodontal and orthodontic variables, incidence outcomes and associated factors, using a dual‐reviewer process with consensus resolution. Due to substantial heterogeneity across the studies, the findings were synthesised narratively. The Robins‐E tool was used to assess the risk of bias and the grade approach was used for certainty of evidence assessment.

**Results:**

Ten studies involving 1246 patients were included in the analysis. The risk of bias was generally of ’some concerns’ and the certainty of evidence ranged from low to very low. The incidence of OGE during orthodontic treatment ranges from 18% to 78%. Among the various associated factors reported, the most frequently statistically significant ones were the distance from the contact point to the crestal bone, crown morphology, crowding and age. Although formal quantitative synthesis was precluded due to clinical and methodological heterogeneity, consistent directional trends across studies, support the relevance of these factors in the development of OGE during orthodontic treatment.

**Discussion:**

The appearance of OGE is relatively frequent during orthodontic treatment. These results could be useful in better understanding and preventing the contributing factors. Further large‐scale, well‐designed prospective longitudinal observational cohorts are required to provide more robust data.

## 1. Introduction

**Figure 1:**
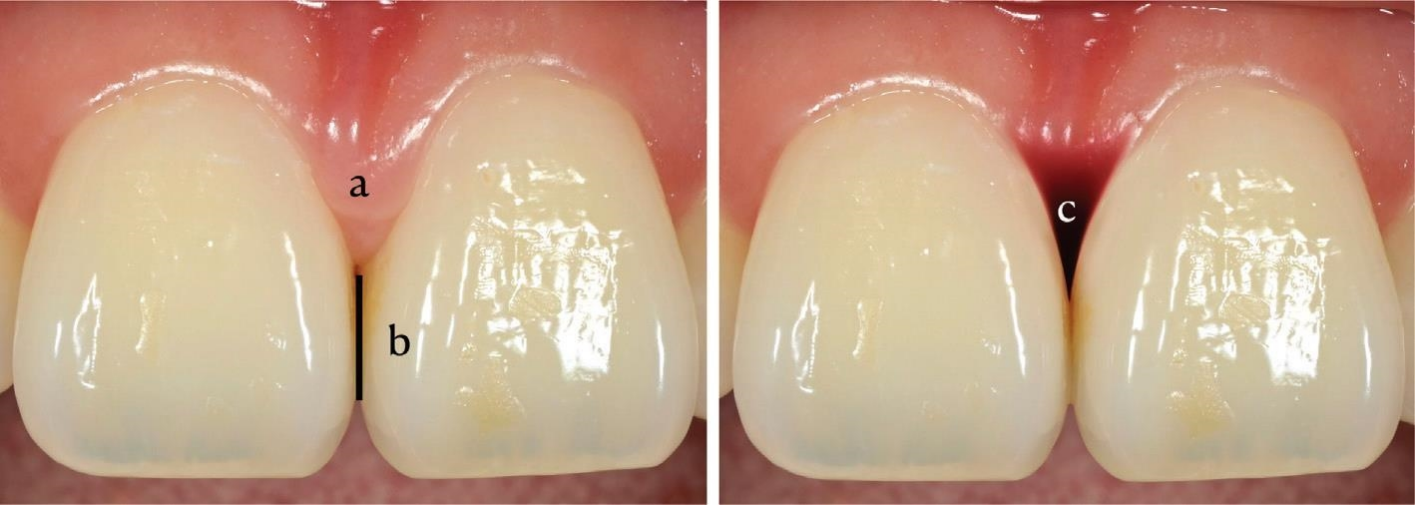
Illustration of open gingival embrasure: (a) Inter‐dental papilla, (b) contact surface and (c) open gingival embrasure (image modified).

Open gingival embrasure (OGE), also known as ’black triangle’, occurs when the interproximal space is not fully filled by the interdental papilla (Figure 1). The interdental papilla is a thin, fragile connective tissue that is supported by the underlying alveolar bone. It occupies the space between two adjacent crowns, extending from the alveolar crest to the proximal contact surface. As such, it is the most visible part of the gingiva, and it plays a crucial role in achieving an aesthetically pleasing smile. In the maxilla, the presence of OGE is one of the features rated most negatively in terms of aesthetic smile perception by professionals and laypeople alike [1, 2]. In the maxillary midline, the interdental area is visible in the vast majority of patients during maximum smile [3] and plays a key role in aesthetics even in low smile lines [4]. The papilla can reduce in size (revealing OGE) following periodontitis or, for unclear reasons, sometimes during orthodontic treatment. OGE has also been observed in periodontally healthy patients and has been associated with non‐periodontal factors such as age [5] and tooth shape [6]. The appearance of an OGE during orthodontic treatment may jeopardise the aesthetic results, and consequently, the patient’s satisfaction.

Patients commonly undergo orthodontic treatment to improve the aesthetic appearance of their smile. However, OGE has been observed following orthodontic treatment in adults [7], frequently causing dissatisfaction among patients [8]. While the evolution of the interproximal gingival marginal level during periodontal treatment has been extensively researched, the effect of orthodontic tooth movement on the papilla, and consequently, on the appearance of OGE, remains unclear. The current literature reports a wide range of incidences of OGE after orthodontic treatment and investigates multiple potential associated factors, making it difficult to grasp a clear understanding [7, 9, 10]. This variability, compounded by heterogeneity in study design, diagnostic criteria and outcome definitions, substantially limits comparability across studies and precludes the formulation of evidence‐based clinical recommendations. Furthermore, despite growing clinical concern, there is no consensus on the relative contribution of patient‐related, anatomical and treatment‐related factors nor on their potential interactions. This represents a critical gap as the inability to reliably predict or prevent OGE undermines treatment planning, informed consent and the long‐term aesthetic stability of orthodontic outcomes. There is also uncertainty surrounding interdental papilla reconstruction management as the published surgical and regenerative approaches have shown limited evidence and short‐term follow‐up [11]. Taken together, these limitations emphasise the need for a rigorous and comprehensive synthesis of the available evidence to quantify the incidence of OGE during orthodontic treatment and to critically evaluate the strength and consistency of the associated factors. Such clarification is essential to inform risk assessment, guide clinical decision‐making and identify priorities for future research. Therefore, a comprehensive synthesis of the available evidence is needed to clarify the incidence of OGE during orthodontic treatment and identify the associated factors.

This systematic review aimed to answer the following focused question: in individuals in permanent dentition, what is the incidence of interdental OGE during orthodontic treatment and what are the associated factors?

## 2. Materials and Methods

The systematic review protocol was developed in accordance with the Preferred Reporting Items for Systematic Reviews and Meta‐Analyses (PRISMA) statement [12]. The study protocol was registered with PROSPERO (CRD420251084329). Our review addressed the following PEO question:

In individuals in permanent and healthy dentition, what is the incidence of interdental OGE during orthodontic treatment and what are the associated factors?

• Population: Individuals with a healthy permanent dentition
• Exposure: All types of orthodontic treatments
• Outcomes:
  1. Incidence of OGE during orthodontic treatment
  2. Associated factors

The primary outcome was the incidence of OGE, defined as the proportion of individuals who developed a visible interdental void during orthodontic treatment. The secondary outcome was to identify factors associated with the occurrence of OGE, including patient‐related factors and treatment‐related factors.

OGE is defined as a visible interdental void occurring when the papilla does not reach the contact surface, assessed clinically or on photographs without restriction of location, number, or classification.

Given the objectives of this review, a PEO (Population, Exposure, Outcome) framework was adopted in preference to a PECO (Population, Exposure, Comparison, Outcome) structure. This decision was driven by methodological and empirical considerations. Specifically, the primary outcome (OGE incidence) is descriptive and does not inherently require a comparator. Furthermore, identifying associated factors relies on observational contrasts rather than a single predefined comparison group.

### 2.1. Information Sources and Search Strategy

A comprehensive literature search was conducted in MEDLINE (via PubMed), the Cochrane Library, EMBASE and Scopus, to identify articles published in English up to July 2025. Combinations of MeSH search terms and text words were used: ((“black triangle ^∗^” OR “interproximal space” OR “interdental space” OR “embrasure” OR “papilla” OR “interdental gingiva” OR “proximal periodontium”) AND (“orthodon ^∗^” OR “malocclusion ^∗^” OR “tooth movement” OR “alignment”))NOT implant.

This search strategy enabled the identification of relevant publications and the development of a comprehensive library of studies.

To supplement the search further, a manual search of the literature was performed by searching the reference lists of the included articles.

### 2.2. Eligibility Criteria

Studies were included in this review if they met the following criteria: (1) patients in permanent dentition (older than 12 years); (2) participants without active or previously treated periodontitis and (3) the incidence of OGE during orthodontic treatment had to be reported.

The following types of studies were considered for inclusion: clinical studies, controlled clinical trials, cohort studies, case‐control studies, retrospective and prospective longitudinal observational studies and cross‐sectional and crossover studies.

Studies were excluded if they included patients with restorations, deciduous teeth, implants, craniofacial abnormalities, or a history of maxillofacial surgery.

### 2.3. Screening Methods

Citations were managed using Zotero v.6.0.37 (Center for History and New Media, George Mason University, Virginia, USA). These were then transferred to Rayyan (https://www.rayyan.ai) to remove duplicates. The screening process was performed by two independent reviewers (M.F. and C.A.). Reasons for exclusion were categorised and recorded. Full‐text analysis was performed on studies that met the inclusion criteria or on studies that provided unclear information in the abstract. If the full text was unavailable, the study authors were contacted for access. Any disagreement arising at any stage of the selection process was resolved through discussion. The inter‐reviewer reliability was registered by calculating the kappa correlation for both screenings.

### 2.4. Data Extraction and Collection

Two independent reviewers (M.F. and C.A.) collected data from each study included in the systematic review. The following data were extracted: citation (author/year); study design; number and gender of participants (male/female); mean age; type of orthodontic treatment and appliances used; average treatment duration; method and classification used to assess OGE; recording method; measured teeth; evaluated factors and statistical significance. If the data were incomplete, corresponding authors were contacted for clarification; if the information could not be obtained, the study was excluded.

### 2.5. Study Risk of Bias and Quality Assessment

This process was performed by two independent reviewers (M.F. and C.A.), with disagreements resolved by discussion. The risk of bias was assessed using the ROBINS‐E tool [13]. This tool provides a structured approach to assessing the risk of bias in observational studies. It specifies the causal effect estimated by the results for each study. It includes seven domains of bias, each of which is addressed using a series of questions designed to gather important information about the assessed analysis. Three judgements are then made: risk of bias, predicted direction of bias and impact of risk of bias on conclusions. Once all seven bias domains have been completed, an overall judgement is made for each of these three considerations. If one of the seven domains was downgraded, the overall bias was also downgraded.

The certainty of the evidence was assessed using the GRADE approach [14], which rates the quality of the evidence for each outcome as high, moderate, low, or very low. The assessment was conducted at the outcome level for (1) the incidence of OGE following orthodontic treatment and (2) the association between OGE and associated factors.

### 2.6. Data Synthesis and Statistical Analysis

All characteristics and results of the study were summarised qualitatively. The strength of the association between the investigated factors and OGE was categorised a priori as strong, moderate, weak, or uncertain using a structured qualitative framework. This classification considered the following: (i) effect estimates derived from multivariate regression models; (ii) consistency of findings across studies; (iii) persistence of associations after adjustment for confounding variables and (iv) robustness and comparability of measurement methods. Factors were classified as: strong if they were consistently significant in multivariate analyses across studies, with a stable direction of effect; moderate if they were supported by mixed or partially adjusted evidence; or weak if associations were limited to univariate analyses or were not retained after adjustment. Those reported in a limited number of studies or showing inconsistent results were considered uncertain.

## 3. Results

**Figure 2:**
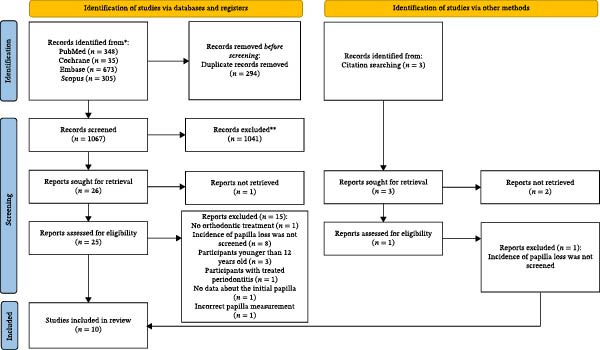
PRISMA flow diagram: Literature search flowchart, adapted from PRISMA 2020. (*Source:* Authors.)

The initial search strategy generated a total of 1361 studies. After removing duplicates, 1067 results were processed for title and abstract screening. Following the exclusion of articles that did not meet the inclusion criteria based on the title and abstract (*n* = 1041), 26 studies underwent full‐text analysis. Data were extracted from 10 papers that met the inclusion criteria using a data extraction form (see Table 1). Any issue or disagreement was resolved through discussion. The identification and selection of articles for this systematic review are summarised in the PRISMA flowchart (Figure 2). Kappa Cohen’s index for screening was 0.915.

**Table 1:** Study characteristics and incidence of OGE.

Study	Country	Study design	Treatment	Type of malocclusion	Treatment duration (months)	Subjects	Site	Papilla classification	Data recording method
Ikeda et al. [15]	Japan	Observational retrospective	FA with PM1 extraction	Class I crowding with no skeletal structural problems	38,22	60	31–41	Ko‐Kimura’s modification of Burke et al. classification	Intraoral photographs, periapical radiographs, study models
Uribe et al. [9]	USA	Observational retrospective	FA with mand incisor extraction	Class I crowding (41), Class II (6) and Class III (4)	NR	51	33–43	Nordland and Tarnow classification	Intraoral photographs
An et al. [16]	South Korea	Observational retrospective	FA without extraction	NR	23.6 6 ± 10.7	100	11–21	Jemt Index	Intraoral photographs lateral cephalograms periapical radiographs
Noverraz et al. [17]	The Netherlands	Observational retrospective	SARME	Transverse maxillary skeletal hypoplasia	33.48 ± 9.12	93	11–21	Jemt Index	Intraoral photographs, CBCT images
Chen et al. [18]	The Netherlands	Observational retrospective	MARPE	Transversal maxillary hypoplasia	17.50 ± 4.27	22	11–21	Jemt Index	Intraoral photographs, CBCT images
Li et al. [10]	China	Observational retrospective	FA with PM extraction	Cl I with moderate or severe crowding	28.88 ± 7.35	39	14–24 and 34–44	Papilla Presence Index	Intraoral photographs
			FA without extraction	Class I with mild crowding	21.6 ± 3.0	43			
Yang et al. [19]	China	Observational retrospective	CA without extraction	NR	26.39 ± 7.67	100	13–23	Nordland and Tarnow classification	Intraoral photographs, panoramic radiographs, digital models
			FA without extraction		25.72 ± 9.71	100			
Zhang et al. [20]	China	Observational retrospective	CA without extraction	Class I (65 patients) Class II (48 patients) Class III (12 patients) 0 mm ≤ anterior crowding ≤3 mm	27.5 ± 12.2	226	11–21 and 31–41	Jemt Index	Intraoral photographs
Cui et al. [21]	China	Observational retrospective	CA without extraction	Class I (28 patients Class II (30 patients) Class III (24 patients)	26.63 ± 6.70	82	13–23 and 33–43	Jemt Index	Frontal and lateral intraoral photographs, panoramic radiographs, digital models
Tian et al. [22]	China	Observational retrospective	CA with and without extraction	Class I, II and III patients	27.58	330	11–21 and 31–41	Jemt Index	Intraoral photographs, lateral cephalograms, CBCT images
			FA with and without extraction						
Total						1246			

Abbreviations: CA, clear aligners; FA, fixed appliance; MARPE, miniscrew‐assisted rapid palatal expansion; NR, not reported; OT, orthodontic treatment; PM, premolar; SARME, surgically assisted rapid palatal expansion.

### 3.1. Study Characteristics

The study characteristics of the included studies are summarised in Table 1. All of the studies that fulfilled the inclusion criteria for our review were retrospective [9, 10, 15–22]. The oldest article was published in 2004 [15] and the most recent articles were published in 2025 [21, 22]. The studies were conducted in six different countries. Four different classifications were used to assess OGE: the Jemt index [16–18, 20–22], the Tarnow and Nordland classification [9, 19], the Papilla Presence index [10] and Ko‐Kimura’s modification of the Burke et al. classification [15].

In total, this review encompasses 1246 participants, mainly individuals aged between 20 and 30 years old, from the set of selected trials.

Four studies [16, 19–21] performed orthodontic treatment without tooth extraction, while Ikeda et al. [15] performed orthodontic treatment involving the extraction of premolars. Two studies [10, 22] involved orthodontic treatment with and without premolar extraction, while Uribe et al. [9] performed treatment involving the extraction of one mandibular incisor. Two studies [17, 18] treated patients with maxillary expansion. Fixed appliances were used in eight studies [9, 10, 12, 15–19] and clear aligners in four [19–22] (two studies used both fixed appliances and clear aligners [19, 20].

All studies took measurements using intraoral photographs; some also used radiographs (periapical, panoramic, CBCT, or lateral cephalometric radiographs), and three studies [15, 19, 21] used study models.

### 3.2. Risk of Bias Analysis

**Figure 3:**
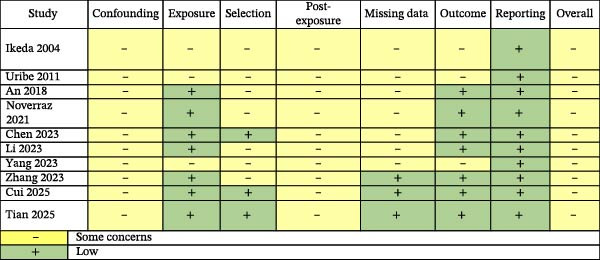
Risk of bias assessment.

The results of the risk of bias assessment conducted using the ROBINS‐E tool are summarised in Table 2 and Figure 3. Overall, all included studies were judged to be at some risk of bias. The main limitations were residual confounding factors and missing data, which reflect the inherent constraints of observational study designs. Exposure measurement, outcome measurement and reporting were generally less problematic and were considered at low risk of bias in the most recent studies.

**Table 2:** Risk of bias assessment.

Study	Bias due to confounding	Bias arising from measurement of the exposure	Bias in selection of participants into the study	Bias due to post‐exposure interventions	Bias due to missing data	Bias arising from measurement of the outcome	Bias in selection of the reported result	Overall bias
Ikeda et al. [15]	Some concerns	Some concerns	Some concerns	Some concerns	Some concerns	Some concerns	Low	Some concerns
Uribe et al. [9]	Some concerns	Some concerns	Some concerns	Some concerns	Some concerns	Some concerns	Low	Some concerns
An et al. [16]	Some concerns	Low	Some concerns	Some concerns	Some concerns	Moderate	Low	Some concerns
Noverraz et al. [17]	Some concerns	Low	Some concerns	Some concerns	Some concerns	Moderate	Low	Some concerns
Chen et al. [18]	Some concerns	Low	Low	Some concerns	Some concerns	Moderate	Low	Some concerns
Li et al. [10]	Some concerns	Low	Some concerns	Some concerns	Some concerns	Moderate	Low	Some concerns
Yang et al. [19]	Some concerns	Some concerns	Some concerns	Some concerns	Some concerns	Some concerns	Low	Some concerns
Zhang et al. [20]	Some concerns	Low	Some concerns	Some concerns	Low	Moderate	Low	Some concerns
Cui et al. [21]	Some concerns	Low	Low	Some concerns	Low	Moderate	Low	Some concerns
Tian et al. [22]	Some concerns	Low	Low	Some concerns	Low	Moderate	Low	Some concerns

### 3.3. Certainty Assessment

Certainty of evidence was assessed using the GRADE framework (Table 3). Because all included studies were observational, the certainty of evidence was initially rated as low and subsequently downgraded where appropriate according to the GRADE domains. The certainty of evidence regarding the incidence of OGE during orthodontic treatment was rated as very low. Although the risk of bias was not considered serious, substantial inconsistency was identified due to wide variation in incidence estimates across studies, reflecting differences in populations, orthodontic treatments and outcome definitions. No serious concerns were identified regarding indirectness, imprecision, or publication bias.

**Table 3:** Certainty assessment (GRADE) of OGE incidence and of associated factors of OGE during orthodontic treatment.

Factor	Number of studies	Study design	Risk of bias	Inconsistency	Indirectness	Imprecision	Publication bias	Other considerations	Certainty
Incidence	10	Retrospective	Not serious	Serious	Not serious	Not serious	Undetected	None	Very low
BC‐CP	4	Retrospective	Not serious	Not serious	Not serious	Not serious	Undetected	None	Low
Crown morphology	4	Retrospective	Not serious	Not serious	Serious	Not serious	Undetected	None	Very low
Crowding	3	Retrospective	Not serious	Not serious	Serious	Not serious	Undetected	None	Very low
Age	6	Retrospective	Not serious	Not serious	Not serious	Not serious	Undetected	None	Low
Tooth movement	2	Retrospective	Serious	Not serious	Serious	Serious	Undetected	None	Very low
Treatment duration	6	Retrospective	Not serious	Not serious	Not serious	Not serious	Undetected	None	Low

The certainty of evidence for factors associated with OGE ranged from low to very low. The risk of bias was generally not considered serious, except for tooth movement, where serious concerns were identified due to confounding and heterogeneous definitions of exposure. No serious inconsistency was detected as the direction of associations was broadly consistent across studies despite differences in statistical significance. Indirectness was judged serious for crown morphology, crowding and tooth movement due to variability in how these factors were defined and measured. Imprecision was considered serious only for tooth movement. Overall certainty was low for age, BC‐CP and treatment duration and very low for crown morphology, crowding and tooth movement.

### 3.4. Incidence

Incidences of OGE during orthodontic treatment reported in the included studies range between 18% and 78% of patients affected.

### 3.5. Associated Factors

**Figure 4:**
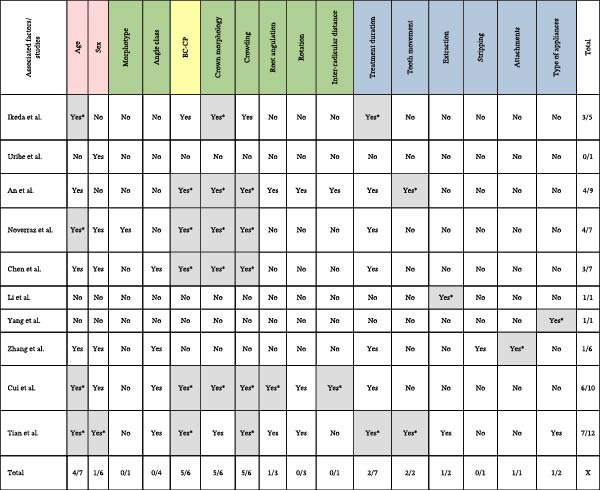
Associated factors.

**Figure 5:**
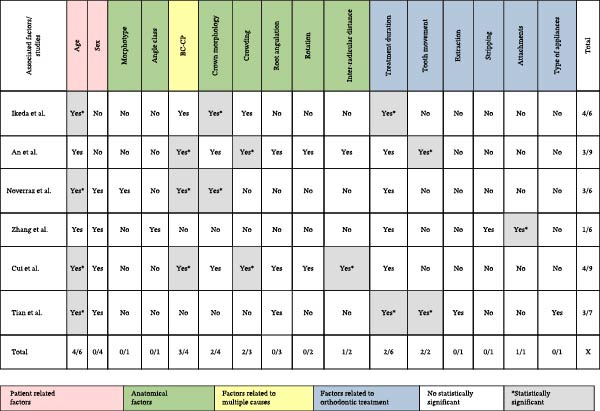
Factors studied in regression models.

The associated factors analysed in each study are summarised in Figures 4 and 5.

Regression models were applied in six studies [15–17, 20–22]. Odds ratios of statistically significant variables in multivariate logistic regression are presented in Table 4. Due to substantial heterogeneity in the examined parameters, measurement methods and analytical approaches, it was not possible to pool the associated factor data from the included studies or calculate cut‐off values or ranges at which these factors become critical for OGE. Therefore, any relationships identified between these factors and OGE should be considered preliminary and require confirmation in future investigations.

**Table 4:** Odds ratios from multivariate analysis.

Associated factors/Studies	BC‐CP		Crown morphology			Crowding		Age	Tooth movement		Treatment duration	Attachments		Sites (maxilla)	
	BC‐CP (variation)	BC‐CP (after treatment)	Crown width	Crown width/length ratio	Mesial‐distal CEJ	A‐P overlap	Crowding	Age	U1 horizontal movement	U1‐SN change	Treatment duration	Anterior	Central (>2)	3–2 vs. 1–1	2–1 vs. 1–1
Ikeda et al. [15] (Reg. Coeff.)			1.233 (0.282)		−1.78 (0.152)			0.053 (0.019)			0.02 (0.009)				
An et al. [16], OR, SE		Maxilla: 1.999(0.31) Mandible: 3.10(0.47)				Maxilla: 2.206(0.37)			Maxilla: 0.84(0.072)						
Noverraz et al. [17], OR, 95%CI	1.50 [1.02; 2.20]			0.91 [0.84; 0.98]				1.10 [1.04; 1.16]							
Zhang et al. [20], OR, 95%CI													1–2: 9.555 (1.157–78.923) >2: 9.578 (1.156–79.368)	9.501 (1.081–83.522)	
Cui et al. [21], OR, 95%CI		Maxilla: 8.046 (4.016–16.122) Mandible: 3.475 (2.390–5.052)		2.400 (1.146–5.027)			Mandible: 1.182 (1.020–1.371)	Maxilla: 1.119 (1.048–1.195) Mandible: 1.068 (1.018–1.121)						0.358 (0.143–0.896)	0.321 (0.122–0.842)
Tian et al. [22], OR, 95%CI								1.157 (1.094–1.224)		Maxilla: 0.929 (0.870–0.991) Mandible: 0.914 (0.805–1.038)	1.036 (1.001–1.071)				

The most frequently statistically significant factors associated with OGE were distance from the interdental contact point to the crestal bone, crown morphology, crowding and age.

#### 3.5.1. Distance From the Crestal Bone to the Interdental Contact Point (BC‐CP)

Six of the selected studies reported on variations in this distance before and after orthodontic treatment [15–18, 21, 22]. With the exception of the Ikeda et al. study, this distance increased significantly more during orthodontic treatment for patients with OGE. Four studies [16, 17, 21, 22] reported the distance between the contact point and the alveolar ridge at the end of the treatment. In all but the Noverraz et al. study [17], this distance was statistically greater for the group with papilla reduction than for the group without papilla reduction. In adjusted analyses, BC‐CP remained associated with OGE in most studies [16, 17, 21] except for one study [15]. Although this association was observed across multiple studies, the certainty of the evidence was low, which limits confidence in this relationship. Accordingly, the strength of the evidence is moderate, reflecting a consistent directionality, but there are substantial methodological limitations (study design and residual confounding).

#### 3.5.2. Crown Morphology

**Figure 6:**
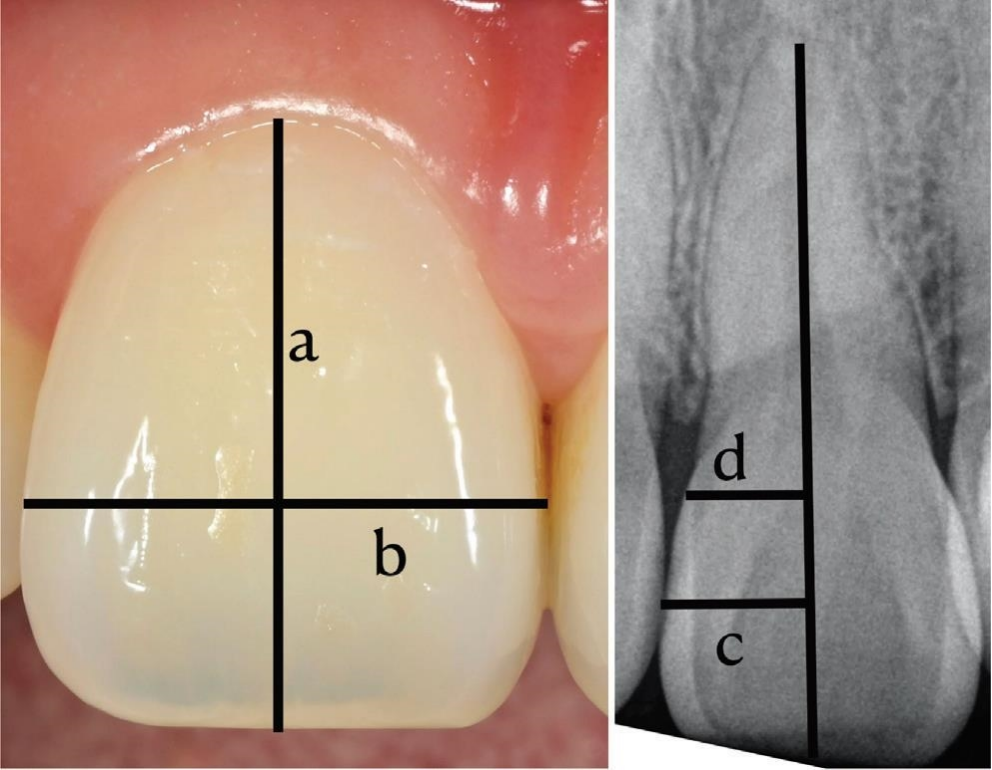
Crown morphology measurements: (a) Crown length, (b) crown width, (c) perpendicular distance from the mesial inter‐dental contact‐point to the tooth long axis and (d) perpendicular distance from the mesial cemento‐enamel junction to the tooth long axis.

A statistically significant difference was found in five [15–18, 21] of six studies [15–18, 21, 22] that examined the impact of crown morphology. Three of these studies [15, 16, 21] evaluated crown shape by measuring crown divergence, which was calculated as the ratio of the perpendicular distance from the mesial cemento‐enamel junction to the interproximal contact point to the tooth long axis (see Figure 6). These studies found that a more divergent (or triangular) crown shape was associated with OGE. The other two studies [17, 18] assessed crown morphology by using the ratio of crown width to crown length (see Figure 6). These studies found that patients with more rectangular and less square crowns were more likely to have an OGE. When assessed using multivariate analysis, crown morphology remained a moderate independent factor in two of the four studies [15, 17]. Variability in measurement methods (e.g., crown ratio vs. geometric divergence) limited direct comparability across studies, resulting in very low certainty of evidence due to indirectness.

#### 3.5.3. Crowding

Six of the 10 selected articles considered the influence of pre‐treatment crowding on OGE [15–18, 21, 22]. Five of them found a statistically significant difference, with greater crowding being linked to a higher frequency of OGE [16–18, 21, 22]. Two of the three studies [15, 16, 21] confirmed this association in an adjusted analysis. Crowding demonstrated a moderate association with very low certainty of evidence due to indirectness arising from heterogeneous definitions of crowding.

#### 3.5.4. Age

Age was the most frequently reported factor (in 8 of the 10 studies). Four of these studies found a statistically significant difference [15, 17, 21, 22]. The older the patient, the more likely he is to develop OGE. Age was identified as an independent factor in four of six multivariate analyses [15–17, 20–22]. Although a generally consistent direction of association was observed, the effect sizes were modest and not consistently retained after adjustment. Increasing age showed a moderate positive association with OGE, although the certainty of the evidence was low.

#### 3.5.5. Other Factors

Many other factors have been linked to OGE with varying degrees of consistency.

Among the studies included in the review, only one [17] reported the periodontal phenotype, highlighting the difficulty in defining the phenotype on photography. The presence of diastema or gingival inflammation was not reported.

The average treatment duration across the studies ranged from 17.50 to 38.22 months across the studies and was weakly correlated with OGE; only two studies found a statistically significant association [15, 22].

The correlation between the type of appliances used and the incidence of OGE was investigated in two studies. Yang et al. [19] found by direct comparisons a statistically significant higher incidence of OGE after treatment in the clear aligner group than in the fixed appliance group (31.5% vs. 21%), whereas Tian et al. [22] found no significant difference between the two groups.

Regarding premolars extractions, Li et al. [10] found a higher incidence of OGE in the extraction group than in the non‐extraction group by direct comparisons (46.2% vs. 25.5%, *p* < 0.05), whereas Tian et al. [22] found no significant difference.

Palatal expansion was realised in two studies [17, 18]. Chen et al. [18] found that the incidence of mild OGE following miniscrew‐assisted rapid palatal expansion (MARPE) was lower than that reported by Noverraz et al. [17] following surgically assisted rapid maxillary expansion (SARME) (18% vs. 32%).

The effect of orthodontic movement on OGE was reported in two studies about incisors [16, 22]. Both concluded that the most consistent movement‐related factor to OGE was lingual inclination of the maxillary incisors. Using a multivariable logistic regression model, An et al. [16] showed that, among multiplanar orthodontic movement, lingual movement of the maxillary incisors was the only independent factor associated with OGE, with each millimetre of lingual retraction increasing the risk by around 18%. Tian et al. [22] corroborated this finding, identifying lingual sagittal movement, especially the lingual movement of the maxillary incisors, as being significantly associated with OGE. Conversely, labial movement appears to have no demonstrable effect. On the mandibular anterior teeth, horizontal tooth movements seem to exert weaker or inconsistent influences on OGE. Lingual movement of the maxillary incisors demonstrated a moderate association with OGE in multivariate models. Greater lingual retraction has been associated with an increased risk of OGE. However, the strength of the evidence remains very low due to limited replication and the potential for residual confounding factors.

Table 5 presents a stratified analysis based on the treatment type and patient characteristics. Both baseline anatomical features and treatment‐related factors may contribute to the occurrence of OGE, although the evidence remains limited and inconsistent. Among patient‐related variables, BC‐CP, crown morphology, crowding and age demonstrated a moderate association. Among treatment‐related factors, the lingual movement of the maxillary incisors was the most consistently reported factor. In contrast, appliance type, extraction protocols and treatment duration showed weak or inconsistent associations. However, due to the low certainty of the evidence, methodological heterogeneity and potential residual confounding factors, the relative contribution and independence of these factors remain uncertain.

**Table 5:** Stratified analysis based on patient characteristics and treatment type.

Domain	Primary factor	Effect on OGE	Patient‐related modifiers	Treatment‐related modifiers	Strength of association	Certainty of evidence
Patient characteristics	BC‐CP distance	Greater distance and increase associated with OGE	‐ Crown morphology ‐ Crowding	‐ Tooth movement magnitude ‐ Contact point displacement	Moderate	Low
	Crown morphology	Higher risk with triangular/divergent crowns	‐ BC‐CP distance ‐ Crowding	‐ Interproximal reduction	Moderate	Very low
	Crowding (pre‐treatment)	Greater crowding associated with higher OGE	‐ BC‐CP distance ‐ Crown morphology	‐ Extraction decisions ‐ Alignment and space closure	Moderate	Very low
	Age	Slight increase of OGE with age	‐ Periodontal condition ‐ Tissue phenotype (unmeasured)	‐ Treatment duration ‐ Movement magnitude	Moderate	Low
Treatment type	Lingual movement of maxillary incisors	Greater movement associated with OGE	‐ BC‐CP distance ‐ Crown morphology ‐ Crowding ‐ Age	‐ Extraction (space closure) ‐ Magnitude of movement ‐ Treatment duration	Moderate	Very low
	Labial movement	No association	None identified	None identified	Uncertain	Very low
	Mandibular anterior movement	Inconsistent	Possibly crowding	Type/extent of movement	Uncertain	Very low
	Treatment duration	Weak association	‐ Age ‐ Baseline severity	‐ Extent of tooth movement ‐ Complexity of mechanics	Weak	Low
	Appliance type (aligners vs. fixed)	Mixed results	Not assessed	‐ Type of movement performed ‐ Case complexity	Uncertain	Very low
	Premolar extraction	Mixed results	‐ Crowding severity ‐ Baseline anatomy	‐ Amount of space closure ‐ Lingual retraction	Uncertain	Very low
	Maxillary expansion (MARPE vs. SARME)	Lower OGE with MARPE (indirect)	Not assessed	‐ Expansion protocol ‐ Subsequent movement	Insufficient evidence	Very low
Unassessed factors	Periodontal phenotype, inflammation, diastema	Unknown	—	Not assessed	Unknown	Very low

The site‐based analysis did not clearly demonstrate distinct differences in OGE incidence across the anterior regions. The incidence ranges substantially overlapped (see Table 6). Due to significant variations in study design, populations, outcome definitions and measurement methods, the observed variations cannot be interpreted as genuine, site‐specific differences. Overall, the available evidence suggests that the incidence of OGE is comparable across anterior sites, and any apparent differences are likely to reflect variability at the study level rather than consistent anatomical effects. Further standardised, site‐specific investigations are required to clarify whether meaningful regional susceptibility exists.

**Table 6:** Site‐based analysis.

Site group	Studies	Treatment types represented	Sample size	Range (%)
Maxillary central (11–21)	An et al. [16], Noverraz et al. [17], Chen et al. [18], Zhang et al. [20], Tian et al. [22]	FA, SARME, MARPE, CA	771	18%–39%
Maxillary extended (13–23)	Li et al. [10], Yang et al. [19], Cui et al. [21]	FA, CA	221	35.4%–78%
Mandibular central (31–41)	Ikeda et al. [15], Uribe et al. [9], Zhang et al. [20], Tian et al. [22]	FA, CA	667	33–68.6%
Mandibular extended (33–43)	Li et al. [10], Cui et al. [21]	FA, CA	125	35.4%–78%
Mixed anterior (both arches)	Li et al. [10], Zhang et al. [20], Cui et al. [21], Tian et al. [22]	FA, CA	720	39%

## 4. Discussion

This systematic review explored the relationship between OGE and orthodontic treatment in periodontally healthy patients. Periodontitis leads to clinical attachment loss and interproximal recession. Since orthodontic treatment does not cause periodontitis, periodontitis cannot be considered an intermediate step in the causal chain from orthodontic treatment to OGE. Rather, it should be considered as an independent cause of OGE. Therefore, periodontitis can be considered an independent risk factor rather than a confounding factor one [23].

OGE during orthodontic treatment has scarcely been studied. The 10 studies included in this systematic review were all retrospective, and there was substantial heterogeneity in terms of study design, patient characteristics, orthodontic protocols and methods of assessing outcomes. Sample sizes varied considerably and various classification systems were employed to evaluate OGE. This variability precludes quantitative synthesis and limits the comparability of findings across studies.

The reported incidence of OGE varied considerably, ranging from 18% to 78%. This wide range is likely to arise from differences in measurement methods, the definitions of OGE, the treatments used and the patient populations studied. Therefore, these estimates should be interpreted with caution.

Among numerous factors reported, our study identified the following as the most frequently associated with OGE during orthodontic treatment: distance from the contact point to the bone crest, crown morphology, age and crowding.

### 4.1. Crestal Bone to Interdental Contact‐Point Distance (BC‐CP)

**Figure 7:**
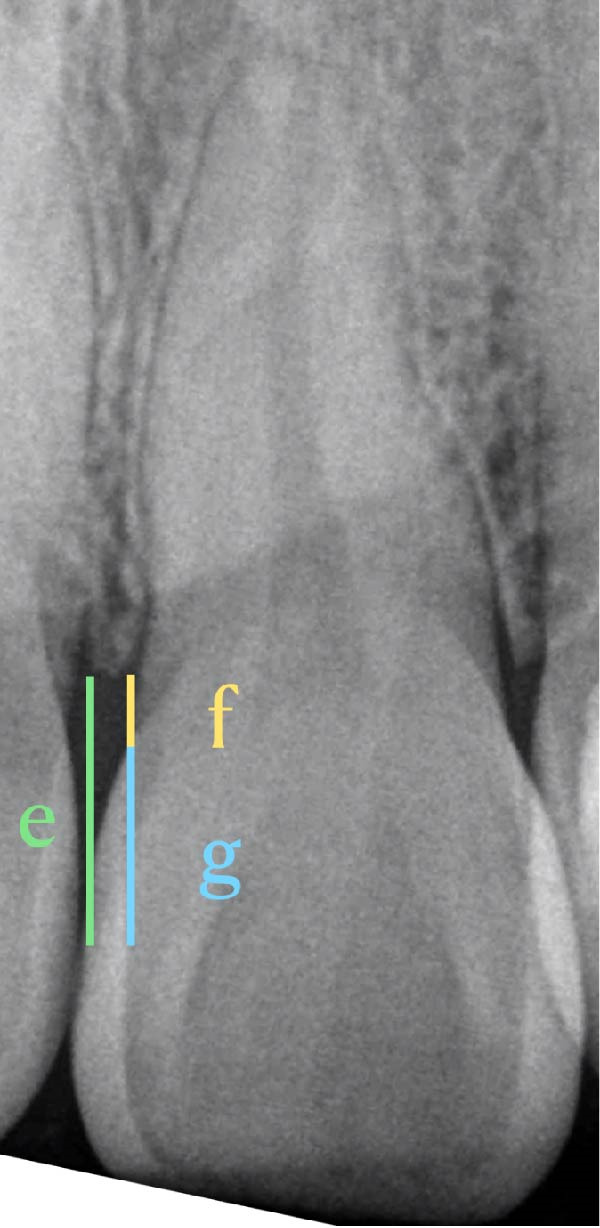
Distance from the crestal bone to the interdental contact point (BC‐CP). (e) Distance from the crestal bone to the interdental contact point (BC‐CP), (f) distance from the crestal bone to the cemento‐enamel junction (BC‐CEJ) and (g) Distance from the cemento‐enamel junction to the contact point (CEJ‐CP); (e = f + g).

The association between BC‐CP distance and OGE has been widely accepted since the highly cited study by Tarnow et al. [24] reported that the papilla was almost always present when the distance was ≤5 mm and almost always absent when the distance was ≥7 mm. These findings were corroborated by subsequent studies [25–28]. The BC‐CP distance can be considered the sum of two components: the distance from the bone crest to the cemento‐enamel junction (BC‐CEJ) and the distance from the cemento‐enamel junction to the contact point (CEJ‐CP) (see Figure 7). In healthy individuals, the BC‐CEJ distance can vary between 1.0 and 3.0 mm [29, 30], and a positive correlation has been observed between the CEJ‐BC distance and papilla height [31]. Chang [28] found that BC‐CEJ was the strongest predictor of papilla presence, indicating that it was a more reliable parameter than BC‐CP for predicting papilla recession. In a subsequent study [32], he also reported a negative correlation between clinically observable papillae and BC‐CEJ: a 1 mm increase in BC‐CEJ corresponded to a 0.46 mm decrease in papilla height.

Various amounts of alveolar bone loss have been reported during orthodontic treatment [33–35]. However, it is unclear whether variation in the bone level during orthodontic treatment affects OGE. Ikeda et al. [15] did not observe a significant difference in BC‐CEJ between the normal and OGE groups. An et al. [16], Noverraz et al. [17], Chen et al. [18] and Cui et al. [21] found that the BC‐CP increased significantly more for patients with OGE than for patients without, but did not indicate which part of this increase was due to a change in the bone crest and which was due to a change in the contact point position.

The interaction of this factor with multiple anatomical and treatment‐related variables complicates its independent interpretation. In particular, it is influenced by embrasure morphology and bone level. Furthermore, it has been reported that bone level and crown shape are linked (teeth with triangular crowns typically exhibit a longer CEJ‐BC and greater papilla height).

### 4.2. Crown Morphology

Of the six studies included in our review that examined the impact of crown morphology on OGE during orthodontic treatment, five identified a significant association [15–18, 21]. In two of the four studies that assessed it in multivariate analysis, it remained a moderate independent factor [15, 17]. More divergent (or triangular) crowns were associated with a higher frequency of OGE.

In the general population, a negative correlation has been reported between the crown width/crown length (CW/CL) ratio and papilla height as well as between contact point height and papilla height. This indicates that slender (tapered) crown forms are associated with greater papilla height, whereas wider (square) crown forms are associated with a lower papilla height. This relationship has been observed consistently across diverse populations, including Asian and Caucasian groups [25, 36–41]. A positive correlation has also been reported between OGE and the CW/CL ratio [5], suggesting that a positive correlation may exist between the initial papilla height and the development of OGE (the higher the papilla, the more likely it is to reduce in height).

Globally, the incidence of OGE appears to be linked to interdental embrasure volume (the larger and taller the embrasure, the more frequent the papilla loss) [37, 42]. This volume is influenced not only by the tooth size but also by the distance of proximal contact (the more apical the contact, the smaller the embrasure) and the divergence of the proximal crown faces. The shape of the adjacent teeth and their mesio‐distal root divergence influence both the contact distance and the divergence of proximal crown faces.

It has been reported that square teeth have a shorter distance between the cemento‐enamel junction and the crestal bone than triangular teeth. This suggests that crown shape may influence both papilla height and the distance from the BC‐CEJ [31].

### 4.3. Crowding

Studies by Chen et al. [18], Noverraz et al. [17], An et al. [16], Tian et al. [22] and Cui et al. [21] found that crowding significantly affects OGE during orthodontic treatment. Studies by An et al. [16] and Cui et al. [21] confirmed this association in an adjusted analysis The following hypotheses can be proposed to explain this observation.

First, severe crowding can close the interproximal embrasure, leaving no space for the papilla to form and reducing the space between the roots. This has been linked to OGE and missing proximal bone: cancellous bone is absent below 0.5 mm of bone width, and only a periodontal ligament remains below 0.3 mm [43].

Second, aligning crowded teeth increases the interdental space (and therefore the interdental embrasure volume), particularly when the contact point is moved vertically and the distance between the roots is modified [26, 44]. Mesio‐distal root divergence results in a coronal contact point location and increased root distance at the bone crest level, whereas root convergence results in an apical contact point location and decreased root distance at the bone crest level.

Thirdly, alignment can increase the distance between the roots to a point at which the probability of the presence of full papillae decreases [45, 46]. Martegani et al. [47] proposed 2.4 mm as the cut‐off point, regardless of the bone‐level distance to the contact point. Cho et al. [27] reported that papillae were not present when the interdental distance exceeded 4 mm. However, other studies have failed to demonstrate that the interdental distance is a significant factor in multivariate analyses when controlling for age and the distance between the interdental contact point and the bone crest [5, 25, 28].

The capacity of a papilla to grow in newly opened interdental spaces or following enlargement of root proximity or reduction of a wide interdental space, is still unclear.

### 4.4. Age

In our research, age was the most commonly reported factor influencing the interdental papilla during orthodontic treatment. Four of the eight studies that evaluated its impact found a statistically significant association and confirmed it as an independent factor in multivariate analyses [15, 17, 21, 22]. Studies have shown that, independently of orthodontic treatment, increasing age is significantly associated with a reduction in interdental papillae [5, 28, 32]. This relationship may be due to age‐related changes in periodontal tissue, such as an increase in the distance from the bone crest to the contact point [28, 32] and thinning of the soft tissue [48]. Large‐scale epidemiological studies corroborate this, showing that interproximal recession increases with age, even in the absence of changes in probing depth, due to progressive attachment loss [49].

The association between age and papilla reduction was not systematically demonstrated statistically in all the studies included in our review. This may be because the sample sizes were too small to achieve statistical significance with regard to the duration of orthodontic treatment.

### 4.5. Other Factors

Various anatomical factors and factors related to orthodontic treatment have been reported, often interacting with each other. For example, the proximity or angulation of roots can affect the location of the interdental contact point and embrasure morphology. Changes in root position due to crowding or tooth movement can alter the distance between roots, thereby modifying the BC–CP [5, 32, 43, 46].

The influence of periodontal phenotype on OGE remains controversial [5, 6, 31, 36, 46, 50]. This may be due to a possible link between periodontal thickness and BC‐CP or tooth shape and to the different methods used to evaluate these factors [31].

In 2022, Rashid et al. [51] conducted a systematic review to investigate the prevalence of OGE after completing treatment with fixed orthodontic appliances, as well as the associated factors and alveolar bone levels. After reviewing five articles [7, 16, 44, 52, 53], they found an incidence ranging between 38% and 58% and concluded that no definitive conclusions could be drawn due to the heterogeneity of the data.

## 5. Limitations of the Review

Our review provides a comprehensive overview of the current literature on the incidence of OGE in various orthodontic situations along with the associated factors. However, the studies included in our review were retrospective. These designs are associated with a relative low level of proof and prospective longitudinal observational cohorts are required to provide more robust data. The interplay of various factors makes the OGE difficult to understand, especially when studied via univariate statistical analysis. A major limitation when interpreting the data collected in our review is the variety of methods used to measure papillary reduction.

The overall risk of bias across the included studies was of some concern. Most of the studies were observational in design and were, therefore, susceptible to confounding factors. While some analyses adjusted for certain variables, residual confounding is likely, particularly given the inconsistent and limited consideration of relevant factors (e.g., crown morphology, crowding and treatment characteristics). Furthermore, confounding by indication is a concern for treatment‐related variables, such as premolar extraction or appliance type, since these decisions are closely related to the severity of the initial malocclusion and the objectives of the treatment. Another important limitation relates to the heterogeneity in measurement methods. Variability in the definition and assessment of key variables (e.g., crown morphology) and differences in outcome assessment may have introduced measurement bias and reduced comparability across studies.

In accordance with GRADE guidance, heterogeneity across studies was evaluated within the inconsistency domain, taking into account differences in outcome definitions, measurement methods, population characteristics and orthodontic treatment protocols. Although variability in effect estimates was observed, this could largely be explained by these methodological and clinical differences rather than unexplained heterogeneity. Notably, the direction of associations for key factors such as age and tooth morphology was generally consistent across studies. Therefore, inconsistency was not considered serious, and no downgrading was applied in this domain. Publication bias could not be formally assessed because comparable quantitative effect estimates were unavailable across studies. Nevertheless, within the GRADE framework, no clear evidence of publication bias was identified. This judgment should be interpreted cautiously, as evidence from orthodontic research suggests that adverse effects are not consistently or systematically assessed or reported, potentially leading to under‐representation of unfavourable outcomes [54]. Therefore, although publication bias was not detected, selective publication and selective outcome reporting, particularly regarding adverse or aesthetic outcomes, cannot be excluded. These limitations should be considered when interpreting the certainty and completeness of the available evidence.

Overall, the evidence does not support robust causal inferences. Well‐designed prospective studies with standardised outcome measures and appropriate multivariable modelling are required. Future studies should use standardised measurements and classifications based on fixed anatomical references, such as the cemento‐enamel junction (e.g., the Nordland and Tarnow classification of OGE [55]) or the contact surface/crown length ratio rather than the crown width/crown length ratio to evaluate crown morphology [31].

### 5.1. Clinical Consequences

Our work highlights that OGE occurs frequently during orthodontic treatment and identifies some associated recurrent factors. The factors associated with OGE during orthodontic treatment were broadly similar to those reported in non‐orthodontic populations. However, this comparison should be interpreted with caution, given the differences in study design, population and measurement approach. From a clinical perspective, these factors can be categorised as follows:

Anatomical or patient‐related factors: initial bone crest to contact point distance, crown morphology, initial crowding and age.

Orthodontic treatment‐related factors: sagittal displacement and lingual inclination of the maxillary incisors, changes in embrasure, contact point position and the distance between roots in relation to alignment and stripping, as well as bone resorption induced by orthodontic displacement.

Furthermore, a better understanding of the causes and associated factors of OGEs in the general population would aid the diagnosis of periodontitis, particularly in its early stages, and could help to reduce the disease’s visible effects. This knowledge could also be useful for restorative interventions in the aesthetic sector, where integration of soft tissue is essential.

## 6. Conclusion

The reported incidence of OGE ranges from 18% to 78% across studies, reflecting heterogeneity in study designs, populations and methods of assessing outcomes. The factors most frequently investigated include the distance from the crestal bone to the contact point, crown morphology, crowding and age. Although current evidence suggests that these factors are associated with OGE, the magnitude, independence and potential interactions of these associations remain insufficiently defined. Furthermore, existing data do not permit a clear distinction between anatomical characteristics and treatment‐related effects, and the contribution of orthodontic treatment to OGE remains incompletely elucidated.

To improve consistency and interpretability of findings, future investigations should adopt standardised, reproducible outcome measures. In addition, consistent methodology for assessing interproximal bone levels and crown morphology is warranted. Well‐designed prospective studies incorporating standardised protocols and robust multivariable analyses are essential to clarify the determinants and underlying causal mechanisms of OGE in the context of orthodontic treatment.

**Nomenclature**
OGE: Open gingival embrasure
PRISMA: Preferred reporting items for systematic reviews and meta‐analyses
PECO: Population exposure comparison outcomes
ROBINS‐E: Risk of bias in non‐randomised studies–of exposures
GRADE: Grading of recommendations, assessment, development and evaluation
SD: Standard deviation
CI95%: Confidence interval at 95%
CBCT: Cone beam computed tomography
BC‐CP: Distance from the crestal bone to the interdental contact point
BC‐CEJ: The distance from the bone crest to the cemento‐enamel junction
CEJ‐CP: The distance from the cemento‐enamel junction to the contact point
CW/CL: The crown width/crown length ratio
SARME: Surgically assisted rapid maxillary expansion
MARPE: Miniscrew‐assisted rapid palatal expansion.

## Author Contributions

**Charles Angioni**: writing – review and editing, writing – original draft, conceptualisation, methodology, investigation, data curation, formal analysis, visualisation, supervision, project administration. **Marwa Fendi**: writing – review and editing, writing – original draft, conceptualisation, investigation, data curation, formal analysis, visualisation, validation. **Zeyad Hassan**: writing – review and editing, visualisation, validation. **Agnès Kamoun**: writing – review and editing, validation, supervision. **Marjolaine Gosset**: writing – review and editing, validation, supervision. **Adrian Brun**: writing – review and editing, validation, supervision, project administration.

## Funding

This research did not receive any specific grant from funding agencies in the public, commercial, or not‐for‐profit sectors.

## Conflicts of Interest

The authors declare no conflicts of interest.

## Data Availability

The datasets used and/or analysed during the current study are available from the corresponding author upon reasonable request.
